# The Effect of Length and Structure of Attached Polyethylene Glycol Chain on Hydrodynamic Radius, and Separation of PEGylated Human Serum Albumin by Chromatography

**DOI:** 10.34172/apb.2021.082

**Published:** 2020-08-05

**Authors:** Parvin Akbarzadehlaleh, Mona Mirzaei, Mahdiyeh Mashahdi-Keshtiban, Hamid Reza Heidari

**Affiliations:** ^1^Drug Applied Research Center, Tabriz University of Medical Sciences, Tabriz, Iran.; ^2^Department of Pharmaceutical Biotechnology, Faculty of Pharmacy, Tabriz University of Medical Sciences, Tabriz, Iran.

**Keywords:** Human serum albumin, Ion-exchange chromatography, PEG-HSA, PEGylation, PEGylated, Size exclusion chromatography

## Abstract

***Purpose:*** This study focuses on the effect of length and structure of attached polyethylene glycol (PEG) chain on hydrodynamic radius (R_h_ ) and chromatographic retention of PEGylated protein. To this aim human serum albumin (HSA) as a standard protein was PEGylated site specifically with mPEG-maleimide.

***Methods:*** Separated PEG_HSA fractions were analyzed by size exclusion and anion exchange chromatography (AExC). The purity of fractions and the relative mobility of PEGylated and native proteins were analyzed by sodium dodecyl sulfate polyacrylamide gel electrophoresis (SDS-PAGE). Hydrodynamic radius was determined based on the retention time of fractions on size exclusion chromatography (SEC), and also according to the previously reported equations.

***Results:*** A linear relation was shown between the molecular weight of attached PEG and R_h_ of PEGylated HSA. No significant difference between R_h_ of proteins modified with linear and branched PEG was shown. In SDS-PAGE, the delaying effect of branched PEG on movement of PEGylated protein was higher than that of linear PEG.

***Conclusion:*** As PEGylated HSA and dimer HSA have almost the same size and in SEC they elute at very close retention times, so in this case ion exchange chromatography (IExC) is more effective than SEC in separation of PEGylated HSA. Branched PEG- HSA showed earlier elution on anion exchange chromatography compared to linear PEG-HSA, that this can explain the different shielding effect of various structures of attached PEGs. The smaller size of PEGylated HSA in compare to the sum of the hydrodynamic radiuses of native HSA and attached PEG could be as a result of shielded attachment of polymer around protein.

## Introduction

PEGylation is the covalent and non-covalent attachment of activated polyethylene glycol (PEG) chain to various biomolecules. PEG is not toxic and nor immunogenic polymer. This technique is introduced as a strategy to eliminate some clinical disadvantages of biopharmaceuticals. PEGylation causes improvement in the pharmacokinetic properties of the biomolecules due to some changes in hydrodynamic radius and electrostatic binding of the biomolecule.^1^

After PEGylation the hydrodynamic radius of the molecule increases which causes reduced renal excretion and increased maintenance time of the conjugates in the body, and thereby allows reducing the dosing frequency of biomolecule in clinic and consequently the patient’s comfort.^2,3^

In order to benefit from these advantages of PEGylation, since its introduction in the 1970s, a variety of therapeutic bio-molecules have been PEGylated.^4-6^ To understand the importance of size (molecular weight) and structure (linear versus branched) of PEGs on physico-chemical properties of the PEGylated biomolecule, at this study site selective polymer (m-PEG-Maleimide) is conjugated to free sulfhydryl group of cysteine, to produce homogenous monoPEGylated human serum albumin (HSA).^7,8^

HSA has a single polypeptide chain and 17 disulfide bridges, and one free cystein residue (Cys 34) in its structure, and has 585 amino acids (66.5 kDa). This protein is the main protein of plasma with concentration of about 3.5–5.0 g/dL, and supplies 80% of the colloid osmotic pressure of plasma (25-33 mm Hg).^9,10^

This protein with the usual dosage of 10 g/dose is the first choice in treatment of some critical illnesses such as hypoalbuminemia that is caused by increased vascular permeability, exogenous albumin is needed to increase its serum concentration in order to balance the albumin concentration between the extra and intra vascular compartments. However, as macromolecules leak continuously into the interstitial space, this expanding effect of albumin is not enough, and so frequent HSA infusion is necessary in order to manage the interstitial edema, and to maintain the demanded blood concentration.^8,11^ As the size of HSA’s molecule has very important role in its retention during the circulation, therefore increased hydrodynamic radius of HSA by PEGylation, makes it an excellent volume expander that can prevent the interstitial edema, and reduce its infusion periodicity.^8,12^

PEG which is biocompatible and non-immunogenic polymer has wide application in protein conjugation,^13^ that as a result the size of protein increases and so the possibility of its leakages decreases.^2,3^

At 2003 Cabrales et al^15^ have produced HexaPEGylated HSA by conjugating the Maleimide Phenyl PEG5K (MPPEG5K) to HSA, and studied their (native and PEGylated HSA) restoring intravascular volume effect. Later at 2009 Suo et al^16^ PEGylated HSA using solid-phase adsorption method in order to prepare homogeneous PEGylated protein. Zhao et al^8^ have studied site specific PEGylation of HSA at Cys-34 with a 20 kDa PEG-maleimide at 2012, and have pegylated HSA N-terminally with 20k Da mPEG-propionaldehyde at 2013, in which free α-amino group of the N-terminal amino acid residue has conjugated with PEG and monoPEGylated HSA has obtained.^11^

At this study HSA was selected as a standard protein and the effect of PEG structure and molecular weight on hydrodynamic radius and retention profile, and on the mobility of PEGylated HSA in IEX chromatography and in poly-acrylamide gel electrophoresis, respectively was discussed.

## Materials and Methods

### Materials

HSA was purchased from Sigma (St. Louis, MO, USA). The activated PEG products (linear PEG with molecular weights of 5, 10 and 20 kDa, and branched PEG with molecular weight of 20 kDa) were obtained from NOF (Tokyo, Japan).

### PEGylation of HSA with various PEG-Mal

PEG-Mal was added to HSA solution (6 mg/mL) in 50 mmol/L phosphate buffer with pH: 6.5, containing 10 mmol/L ethylene diamine-tetra-acetic acids (EDTA). Protein to polymer molar ratio of 1:3 was used. The reaction mixture was incubated at 37°C for 20 hours in mild shaking condition. To slow down the reaction rate after 20 hours the mixture was kept in refrigerator.

### Purification of the PEGylated HSA

The PEGylated albumin was separated from the unreacted protein and unreacted PEG-Mal, using anion exchange chromatography and size exclusion chromatography (SEC).

IExC was done on TSK-gel Q-STAT (7 µm, 4.6 mm ID ×10 cm L) column from Tosoh (Tokyo, Japan) at room temperature. The reaction mixture was applied to the column and washed thoroughly with buffer A (10mM Tris base, pH: 8 with 0.1 mol/L NaCl) over 5 column volume. Various conditions with different salt gradient methods (linear, step, and mixed linear and step) were examined to achieve optimum resolution of peaks. At this article only the results of optimized method are reported.

SEC was carried out on TSK-gelG3000SW_XL_ (5 µm, 7.8 mm ID ×30.0 cm L) column from Tosoh (Tokyo, Japan) and 0.2 mL reaction mixture was applied on it. 50 mM phosphate buffer with 100 mM NaCl, pH: 6.5 was used as running buffer in isocratic elution with flow rate of 0.8 mL/min. Peaks corresponding to mono-PEGylated protein and unreacted HSA were separately fractionated and concentrated by ultrafiltration with 30 kDa Amicon filters. Fractions from SEC column were anaylzed by 10% sodium dodecyl sulphate–polyacrylamide gel electrophoresis (SDS-PAGE) and IEX chromatography, and vice versa fractions from IEX chromatography column were analyzed by 10% SDS-PAGE and SEC.

### Purity identification of fractions

The purity of fractions from SEC and IExC were analyzed by SDS-PAGE. A discontinuous electrophoresis system consisting of a stacking gel with 5% (w/v) acrylamide and a separating gel with 10% (w/v) acrylamide was employed. Dimensions of the gel were 13.5 cm×13.5 cm×1mm. 30 µL of samples (Reaction mixture and fractionated peaks (from SEC and IEX)) were mixed with 15 µL of loading buffer containing 0.05 mol/L Tris-HCl, 2% SDS, 25% glycerol, 5% 2-mercaptoethanol and 0.01% bromophenol blue, pH 8.8, and incubated at 100°C for 5 minutes. 15 µL of each mixture was loaded in wells. Running buffer was containing 0.1% SDS, 0.025 mol/L Tris base and 0.19 mol/L glycine buffer, pH 8.3. A constant voltage of 110 V was applied. The gel was stained with Coomassie Brilliant Blue G-250 overnight.

### Determining the hydrodynamic radius of pegylated HSA

A calibration curve was created in SEC column applying four standard proteins with known molecular weights including ribonuclease A (13.7 kDa), Ovalbumin (43 kDa), HSA (66.5 kDa), Conalbumin (75 kDa). PBS running buffer at 0.8 mL/min flow rate, and UV absorbance at 280 nm was used to determine the elution volume of each standard protein. Dead volume of column was calculated by elution volume of dextran-blue which was 5.28 mL. K_av_ values were calculated using equation [1].^17^

k_av_ =v_e_ − v_0_ / v_c_ − v_0_ [1]

Hydrodynamic radius calculated using equation [2, 3, 4, and 5]^18^ and fitting calibration standard data.

R_h_=(0.82±0.02)M^1/3^_r_ [2]

RhPEG =0.1912M_r_^0.559^ [3]

[4] $${\text{R}}_{h,PEGprot}\text{ =}\frac{A}{6}+\frac{2}{3A}R_{h,PEG}^{2}+\frac{1}{3}{\text{R}}_{h,PEG}\;$$

[5] $${\text{A=[108R}}_{h,prot}^{3}{\text{+8R}}_{h,PEG}^{3}{\text{+12(81R}}_{h,prot}^{6}+12{\text{R}}_{h,prot}^{3}{\text{)}}^{\frac{1}{2}}{\text{]}}^{\text{1/3}}$$

## Results

Fractionation of the reaction mixture of the HSA PEGylation, yielded three major peaks representing PEGylated HSA, monomer and dimer-HSA. PEGylated HSA was purified from non-PEGylated protein and un-reacted PEG-Mal by IExC and SEC.

### The effect of PEG size (molecular weight) on retention time of PEGylated protein on SEC and IExC

**Figure 1:**
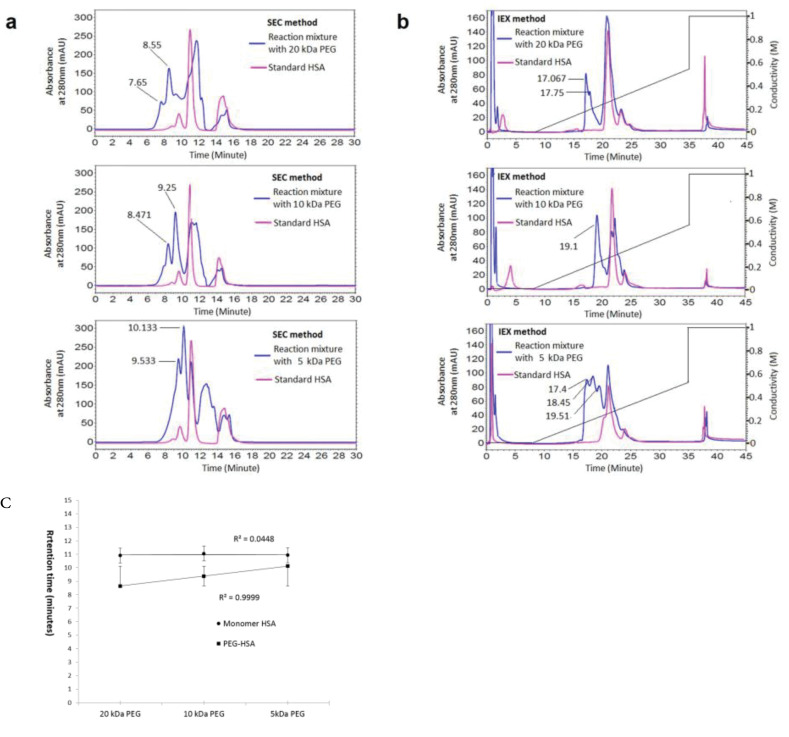
The effect of PEG size (molecular weight) on retention time of PEGylated protein on SEC (a) and IExC (b), (c) is representing the linear increase in retention time of pegylated fraction in SEC versus PEG size.

The results of SEC and IExC separation of PEGylation reaction almost 24 hours after PEGylation by various PEG sizes (5, 10, and 20 kDa) has summarized at Figure 1a and Figure 1b. The retention time of PEGylated form increases linearly on SEC by decreasing the PEG size. The curve of this change and the data of retention times have shown at Figure 1c.

### The effect of PEG structure (linear versus branched) on retention time of PEGylated protein on SEC and IExC

**Figure 2:**
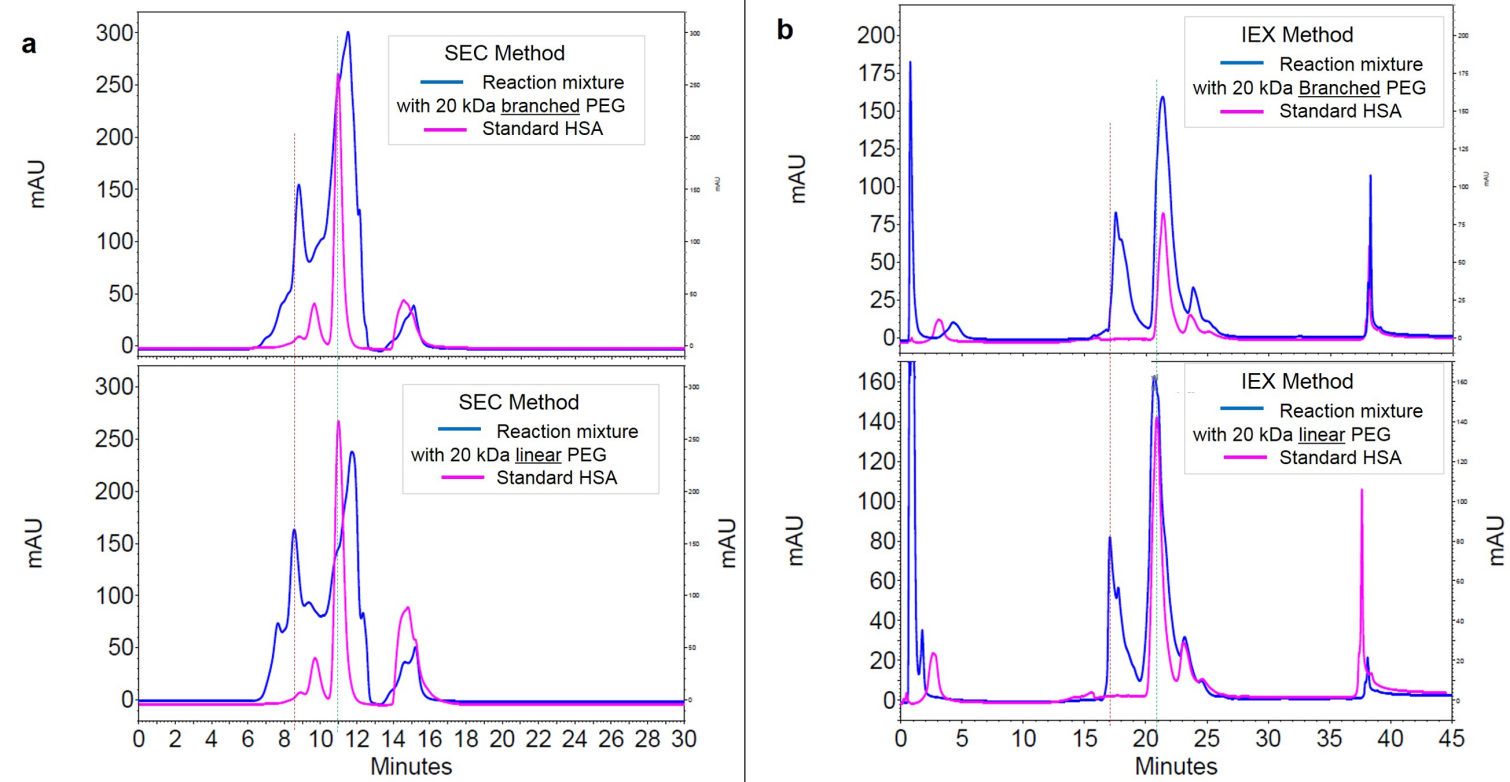
The effect of PEG structure on retention time of PEGylated protein on SEC (graph a at left) and IExC (graph b at right).

The results of SEC and IExC separation of PEGylation reaction almost 24 hours after PEGylation by linear and branched 20 kDa PEG has summarized at Figure 2a and Figure 2b. The retention time of PEGylated fractions has not significant change by various structures of PEG.

### Analysing the fractions of reaction mixture of PEGylation, has done by various molecular weights of linear mPEG-maleimide, in IExC and SEC

**Figure 3:**
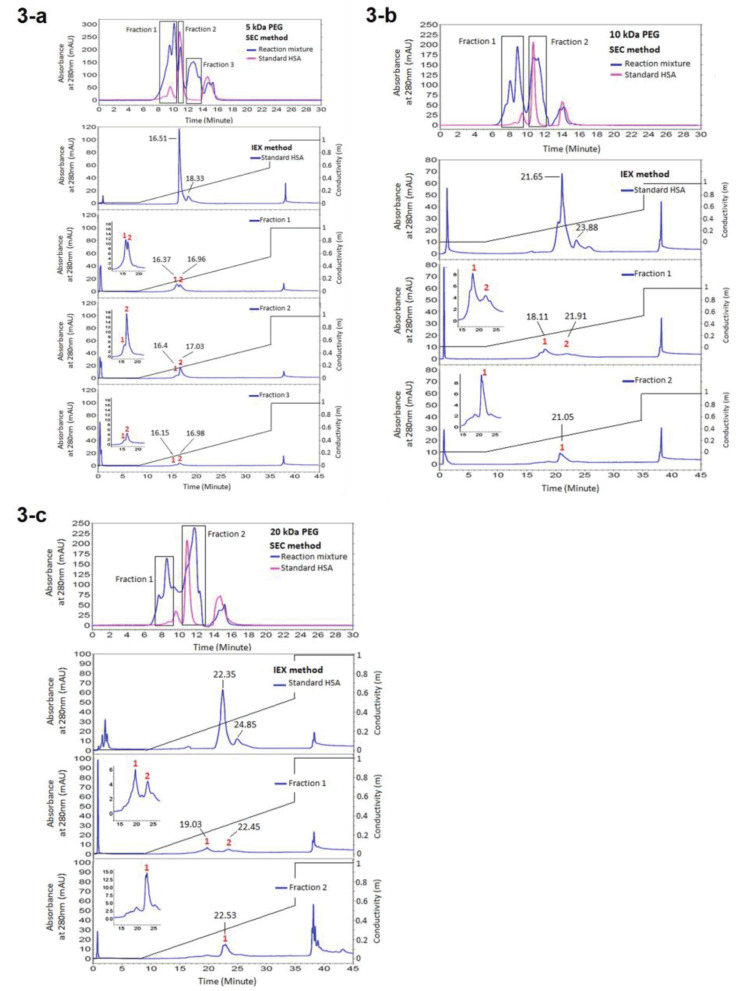
Analysing the fractions of reaction mixture of PEGylation by various molecular weights of linear mPEG-maleimide achieved from SEC, in IEXc. (a) Representing the results of PEGylation by 5 kDa PEG, (b) Representing the results of PEGylation by 10 kDa PEG, (c) Representing the results of PEGylation by 20 kDa PEG

**Figure 4:**
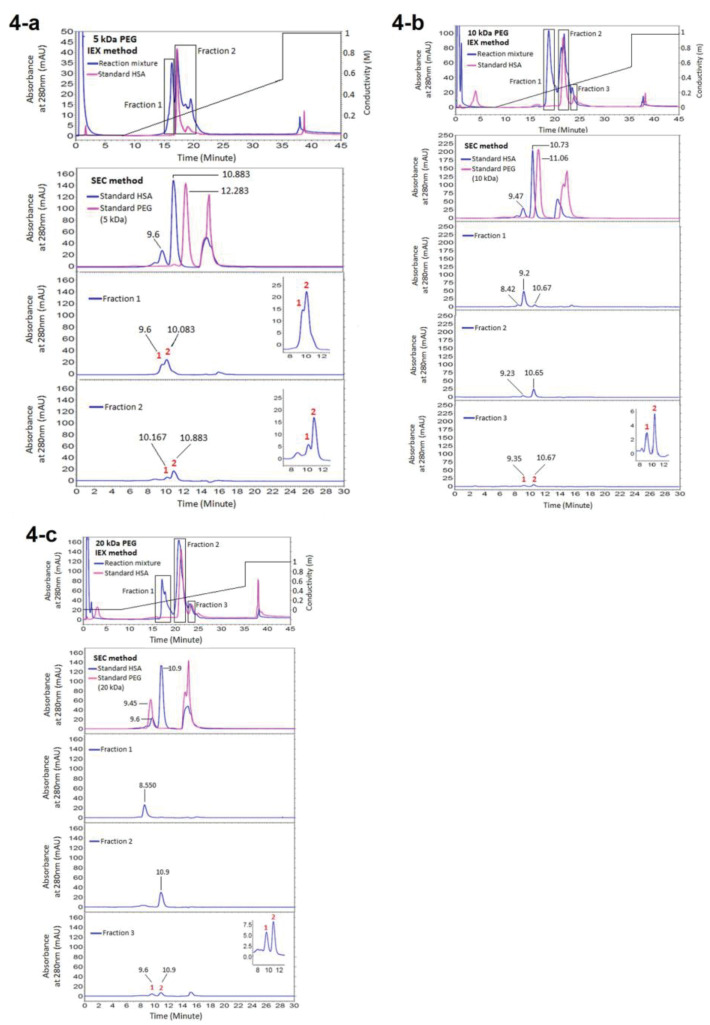
Analysing the fractions of reaction mixture of PEGylation by various molecular weights of linear mPEG-maleimide achieved from IEXc, in SEC. (a) Representing the results of PEGylation by 5 kDa PEG, (b) Representing the results of PEGylation by 10 kDa PEG, (c) Representing the results of PEGylation by 20 kDa PEG

Fractionation of the HSA PEGylation reaction mixture after 24 hours incubation at room temperature yielded three major peaks representing PEGylated HSA, monomer and dimer-HSA. The PEGylated and native form of HSA has fractionated in SEC and IExC and has analysed in IExC and SEC respectively. All parameters which may influence retention time (t_R_) of proteins, including temperature, system pressure and pH of water used for buffer preparation were controlled and kept stable during the experiment. The results are reported at Figures 3 and 4.

**Figure 5:**
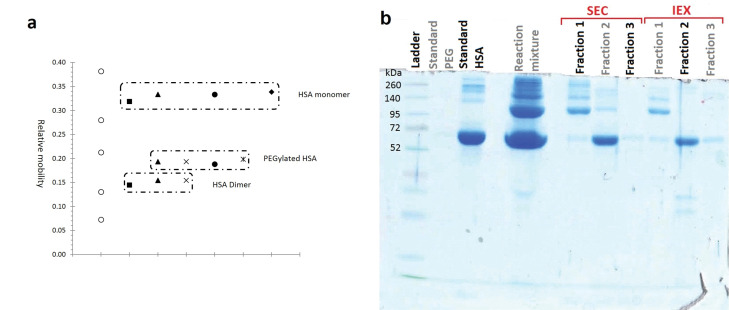
Analysing the purity of fractionated moieties of HSA PEGylation by 20 kDa linear mPEG maleimide

According to the obtained results (Figure 3, Figure 4, Figure 5a, and Figure 5b) purification of PEGylated form of HSA is more efficient in IExC than SEC. In IExC dimer HSA elutes after monomer while pegylated fraction elutes before monomer.

a: Relative mobility of native and PEGylated HSA against their molecular weight (PEGylation by 20 kDa linear mPEG maleimide), b: SDS-PAGE analysis of mixture and separated fractions of HSA PEGylation by 20 kDa linear mPEG maleimide

### Purity analysis

**Figure 6:**
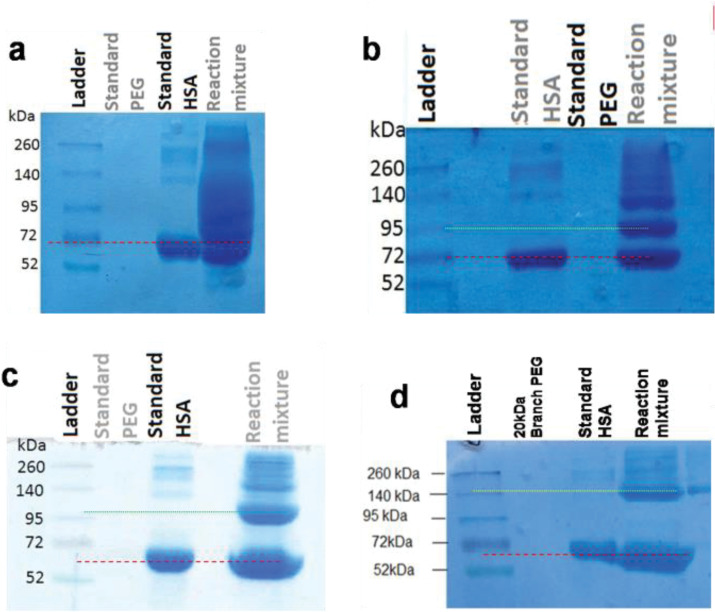
SDS-PAGE analysis of reaction mixture of HSA PEGylation by various PEG sizes and structures. Dash line shows the location of standard HSA and dotted line shows the location of PEGylated HSA compared to ladder.

Results of analyzing the purity of fractionated moieties of PEGylation by 20 kDa linear mPEG maleimide are summarized at Figure 5, and the results of SDS-PAGE analysis of HSA PEGylation by using various PEG size and structure are reported at Figure 6.

a: 5 kDa linear mPEG maleimide, b: 10kDa linear mPEG maleimide, c: 20 kDa linear mPEG maleimide, d: 20 kDa branched mPEG maleimide

By increasing the PEG size from 5 to 20 kDa, the relative mobility of PEGylated fraction decreases. Also Branched structure causes more decrease in relative mobility of PEGylated fraction in compare to linear PEG having the same molecular weight.

As has shown at Figure 5a PEGylated form has relative mobility less than a standard protein with Mw equal to 95 kDa, while the molecular weight of PEGylated HSA is approximately 85.5 kDa.

### Hydrodynamic radius determination

In order to define the hydrodynamic radius (R_h_) of PEGylated and native proteins, a calibration curve has drawn by plotting the K_av_ value (equation1) against molecular weight of standard proteins in SEC on TSK-gelG3000SW_XL_ (5 µm, 7.8 mm ID ×30.0 cm L) column. According to the k_av_ of PEGylated and native fractions of HSA, their molecular weight has estimated.

**Figure 7:**
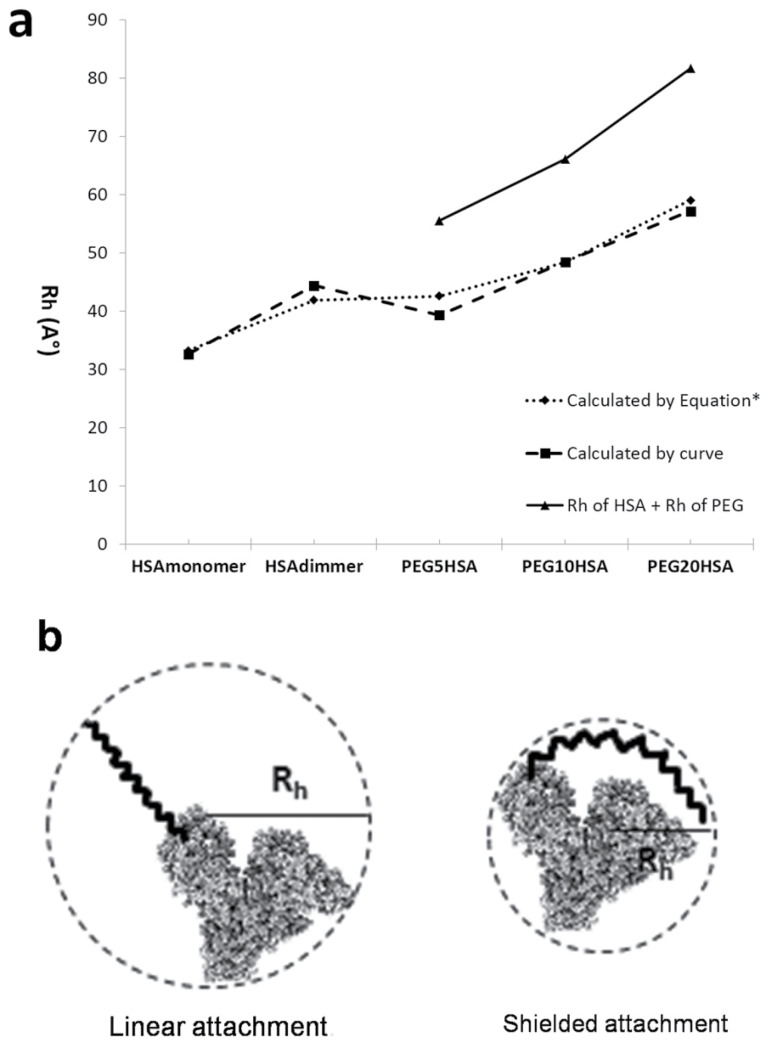
a: Comparing the size of native and PEGylated HSA (with various attached PEGs). Solid line shows the sum of R_h_ value of HSA and attached PEG. Dotted line shows the Rh value calculated by equation, and dashed line shows the R_h_ value calculated by SEC curve. b: Schematic figure to represent the shielded attachment of PEG to protein (right) versus linear attachment (left).

The calculated R_h_ of PEG, PEGylated HSA and native HSA by using the equations 2-5 and also by using the Mw obtained by calibration curve are summarized at Table 1. The size of native and PEGylated HSA (with various attached PEGs) has shown in Figure 7a. The size of PEGylated protein doubles linearly by doubling the molecular weight of attached polymer, and also the sum of R_h_ of attached PEG and HSA is higher than the R_h_ of PEGylated HSA (Figure 7b).

**Table 1:** hydrodynamic radius (size) of pegylated HSA by various PEGs

**Protein**	**K**_av_	**M**_w_ ** (kDa)**		**R**_h_ ** (A˚) of protein by nominal M**_w_		
	**Obtained in SEC**	**Calculated using curve**	**Sum of** **nominal M**_w_	**Calculated using** **equations**	**Calculated using curve**	**Sum of R**_h_ **of HSA and PEG**
HSA monomer	0.382	63.011	66.5	33.22±0.81	32.63±0.8	-
HSA dimer	0267	158.715	133 (66.5*2)	41.86±1.02	44.36±1.08	-
PEG20-HSA	0.172	339.26	86.5 (66.5+20)	59.03±0.57	57.19±1.39	81.72±0.81 (33.22+48.50)
PEG10-HSA	0.234	206.34	76.5 (66.5+10)	48.35±0.53	48.45±1.18	66.14±0.81 (33.22+32.92)
PEG5-HSA	0.312	110.20	71.5 (66.5+5)	42.57±0.76	39.313±0.96	55.57±0.81 (33.22+22.35)

SEC: size exclusion chromatography, HSA: human serum albumin, PEG: polyethylene glycol

**Figure 8:**
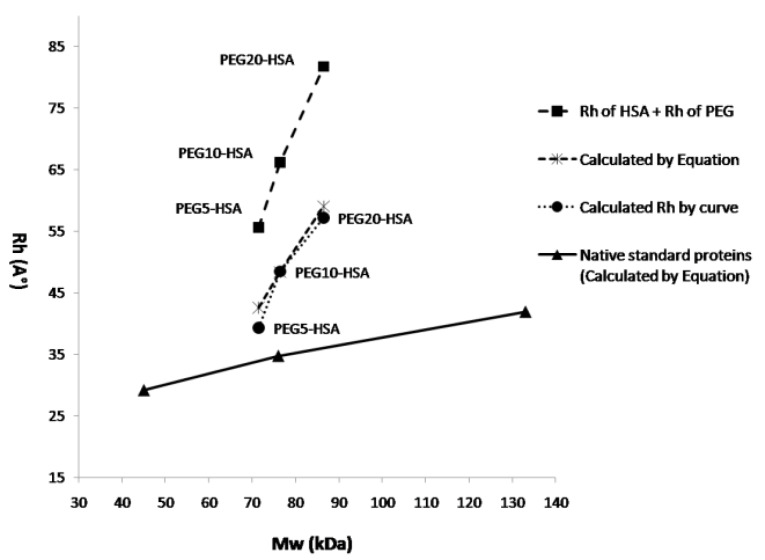
Hydrodynamic radius of PEGylated HSA (with various attached PEGs) and native standard proteins versus their nominal molecular weight. Long dash line shows the sum of R_h_ value of HSA and attached PEG. Dotted and dashed lines show the R_h_ value of PEGylated HSA (with various attached PEGs) calculated by SEC curve and equation respectively, and solid line shows the R_h_ value of native standard proteins calculated by equation 2.

Figure 8 shows the hydrodynamic radius of native standard proteins and PEGylated HSA (with various attached PEGs) versus their molecular weights. The graph shows linear increase in size by increasing the molecular weight of attached PEG. Slope of increase in size for PEGylated forms by increasing the molecular weight of attached PEG, is much sharper than that of native proteins. Also the graph compares the size and Mw of PEGylated forms of HSA with native proteins that when they almost have the same molecular weights, the size of PEGylated form of HSA is much bigger.

## Discussion

PEGylation technique showed rapid increase since its introduction at 1970, because of its major and very useful advantage in increasing the size of protein and consequently increasing the half life of protein in the body. Two factors can affect the hydrodynamic radius of PEGylated protein: the extent of PEGylation and the nature (molecular weight, size) of attached PEG.^13^

In the case of medicines like HSA that have to be used in high dose (usual dosage of 10 g/dose) at various critical hypo-albuminemia conditions (in shocks, burns, trauma, and critical illnesses that vascular permeability increases1-4), and on the other hand have the limited source to obtain (donated blood plasma in the case of HSA), it would be very important to decrease the administration dose by increasing its half life.

In multi-PEGylation methods, which target amine groups, it is difficult to control the PEGylation extent and site of attachment due to the presence of 58 lysine amino acids on HSA. Therefore, site-specific mono-PEGylation methods are desirable to generate proteins that has minimal effects on receptor binding or substrate recognition. While the attachment of maleimide PEG to cysteine has some advantages such as being easy to perform,^19^ in order to provide site-specific attachment, it’s necessary that the protein to have only one free cysteine, as the HSA has at cys-34.

In previous studies PEGylation of HSA has studied from various points of views and with various PEGylation teqniques.^20^

As Zhao et al reported, PEGylation at the only free thiol group on HSA that belongs to Cys-34 results in homogenous mono-PEGylated HAS.^8,21^ Maleimide group is selective for reaction with the reactive thiol group by using the advantage of thiol addition to the activated double bond, forming stable thioether bond, which is known as the Michael reaction.^22^

At this study free thiol PEGylation was carried out at pH 6.5 because the reactivity of the maleimide groups towards thiol group is about 1000-folds faster at this pH than that of primary amines,^23^ consequently, the selectivity to thiol group is higher. Moreover, maintaining the pH slightly below 7 helps to keep HSA in conformation that shields its internal disulfide bonds from other solutes.^24^

The location of terminal amino groups of aromatic amino acids which often is in hydrophobic pockets, or their involvement in hydrophobic interactions, prevent their direct PEGylation.^25^ When the cysteine amino acid of proteins is available in the reduced form, it is usually protected inside cavities that prevent Cys-Cys mediated protein dimerization,^26^ thereby PEGylation at Cys-34 could also be helpful to prevent the heterogeneity in albumin.^8^ Crystallographic studies of albumin show that Cys-34 is buried in a shallow crevice, 9.5 Å deep, while the hydrodynamic radius of 5 kDa, 10 kDa and 20 kDa PEG polymers are 22.3 Å, 32.9 Å and 48.5 Å, respectively.^27^ Limited accessibility of hydrophilic molecules with large hydrodynamic sizes to Cys-34 on HSA, may explain why the modification yield at Cys34 was about 60% and not more.

In the present study thiol group of Cys-34 is targeted for PEGylation and the effect of the size and structure of attached PEG on hydrodynamic radius and purification parameters of PEGylated and native HSA on SEC and IExC has evaluated.

The obtained results at Figure 1 are indicating the effect of molecular weight of attached PEG on retention time of PEGylated fraction, that as has shown at Figure1c there is a linear relation, and t_R_ decreases by increasing the molecular weight of attached PEG in SEC.

Figure 2 represents the effect of structure of attached 20 kDa PEG (linear versus branched) on elution behavior of PEGylated fraction. According to the results, there is no significant difference in SEC. In IExC slightly increase in t_R_ of PEGylated fraction when branched PEG was attached has shown, that this late elution could be due to different shielding and/or steric hindrance imposes by branched PEG to HSA.

From obtained results of analyzing the SEC fractions in IExC, summarized at Figure 3, early eluting peak (fraction1) might be PEGylated HSA as it has bigger size in SEC, the second fraction (fraction 2) belongs to monomer and dimer HSA as it matches with native HSA which was applied as standard. The later peak was noise.

In chromatograms of ion exchange chromatography of reaction mixture of PEGylation to analyze the IExC fractions in SEC (Figure 4), a new peak with lower retention time in addition to peaks of standard native HSA has been produced which indicates production of a derivative with lower interaction with ion exchange matrix as a result of steric hindrance and/or reduced surface charge due to the shielding effect of neutral molecules of PEG^28,29^ that this peak is PEGylated HSA. Second peak and the last peak were monomer HSA and dimer HSA respectively, as they matches with monomer and dimer of native HSA applied as standard.

As previously has reported by Yamamoto et al,^30^ IExC could separate PEGylated fractions more specifically than SEC. In the case of PEGylated HSA, because of the size similarity of PEGylated HSA with dimer HSA, by purification in SEC that both dimer HSA and PEGylated HSA elute before monomer HSA, either purity or yield should be sacrificed, but in IExC dimer elutes after native monomer HSA, while PEGylated fraction elutes before native HSA, and so purification and fractionation of pegylated HSA could be done more efficiently and by more purity.

SDS-PAGE was used to examine the distribution and the molecular weight of PEGylation products. The appearance of an extra band in SDS-PAGE analyses of reaction mixtures comparing to standard HSA (Figure 5 and Figure 6), confirms the formation of PEGylated HSA with a higher molecular weight that travels slower in the gel. Figure 5a compares the relative mobility (R_f_) of PEGylated and native HSA alongside of standard proteins in ladder.

We observed that (Figure 5a) the PEGylated HSA (Mw ≈ 85.5 kDa) migrated faster slower than of a standard protein with molecular weight equivalent to the sum of the molecular weights of HSA and PEG (Mw ≈ 85.5 kDa). This decreased electrophoretic mobility that can be explained by the interaction of SDS and PEG, had been previously reported by Kurfürst.^31^ It has been reported that PEG and SDS may form some kind of a complex, and interfere the SDS-PAGE analysis, resulting in broad, not clear and even smeared bands, that as has reported significantly at the results of PEGylation by 5kDa PEG at Figure 6a. The interaction may happen between PEG chains (either free or coupled to protein) and SDS micelles, and/or between PEG chains and protein, and/or between the PEGylated protein and SDS micelles.^31,32^

On the other hand, as Odom et al have reported, under standard SDS-PAGE conditions, the addition of PEG changes the mobility of the proteins by forming a complex between PEG and the negative charge of SDS micelles. This complex interferes with local conductivity to make proteins ahead of it to move slower, and those behind it to move faster. Changes in the concentration of SDS or PEG can influence the mobility of free proteins.^33^

Comparison of lane corresponding to PEGylated HSA in all cases of HSA PEGylation using different PEG sizes (Figure 6) indicates that as the molecular weight of conjugated PEG decreases, the mobility of PEGylated HSA increases and the band of modified HSA gets closer to un-modified HSA. Because of this, bands of PEGylated HSA with 5 kDa PEG are smeared, which is in agreement with the finding of Zheng et al.^32^ Considering the presence of a large amount of un-reacted PEG especially in the case of PEGylation with 5 kDa PEG which was with 40 folds molar excess, and according to the above mentioned description, resulted smeared bands in SDS-PAGE gel is reasonable.

Results of electrophoresis indicate that fractions have been separated with a satisfactory purity, except for PEGylation by 5 kDa PEG as has explained above.

We calculated, hydrodynamic radius by using equations proposed by Fee and Van Alstine,^21^ and using equations resulted from the calibration curve (Table 1). The experimentally calculated hydrodynamic radiuses (using calibration curve) are in reasonable agreement with hydrodynamic radiuses predicted according to equations. On the other hand, obtained retention times in SEC is very close to expected retention times based on molecular size, which indicates accurate fractionation.

Data in Table 1 show significant increase in hydrodynamic radius of monomer HSA after PEGylation. While the molecular weight of PEG-HSA was just 1.08, 1.15, 1.30 and 1.30 times that of native HSA, the hydrodynamic radius was 1.20, 1.48, 1.75 and 1.83 time of that of native monomer HSA respectively for 5, 10, linear 20 and branched 20 kDa attached PEG (the values of R_h_ are calculated using calibration curve in SEC). The conjugation of HSA with mPEG dedicates the HSA molecule with a great hydrated volume. Due to binding each ethylene oxide unit of PEG to 2-3 water molecules by hydrogen bonds, the size of the PEG molecule becomes 5 to 10 times larger than a protein of comparable molecular weight.^2,3^

PEGylation of HSA with 20 kDa PEG leads to 1.75 folds increase in hydrodynamic radius. Considering retention time of PEGylated albumin in SEC, PEGylated HSA acts as a globular protein with approximately 3.92 times higher molecular weight. In other words, PEGylated HSA with 20 kDa PEG is equivalent in size to an approximately 339.26 kDa native globular protein, which is due to the ability of PEG to coordinate 3 water molecules per oxyethylene monomer. In the same explanation PEGylated HSA with 10 kDa PEG is equivalent in size to an approximately 206.34 kDa native globular protein, and PEGylated HSA with 5 kDa PEG is equivalent in size to an approximately 110.2 kDa native globular protein (Table 1).

Based on obtained results as it has shown in Figure 7a, there is a linear relation between increasing the PEG size and hydrodynamic radius of PEGylated protein. In addition this figure shows that the calculated hydrodynamic radius of PEGylated HSA (dashed line and dotted line) is less than sum of hydrodynamic radiuses of native HSA and PEG (solid line), therefore, it can be concluded that the polymer does not attach to protein in linear form, but as PEG is a flexible molecule it shields the HSA. Figure 7b shows schematically this kid of attachment. Also in agreement with this conclusion Fee and Van Alstine^21^ has declared that at PEG-protein conjugates after a while and during physical events of SEC, in order to keep the surface to volume ratio they normally have in solution, the PEG polymers spreads over the surface of the protein. This helps to rationalize how mono-pegylation of proteins affects prominently both its size (e. g. SEC) and surface properties.

Kwon et al^34^ proposed that, to a first estimation, total hydrodynamic radius of protein is conserved after PEGylation, so its viscosity radius could be calculated as the radius of sphere with the same volume as the sum of the hydrodynamic volume of original native protein and PEG chain. Based on their data, viscosity radius is simply predictable in this way with similar accuracy to equation of Fee and Van Alstine.^21^ PEGylation of small protein with a relatively large amount of PEG chain, results little difference in the viscosity radius calculated by two methods. However, PEGylation of a large protein, particularly with a relatively small amount of PEG chain, the difference will be significant, because according to Fee and Van Alstine^21^ the PEGs outer surface area increase to cover the protein and the hydrodynamic volume must increase corresponding to maintain a constant surface area to volume ratio. Therefore, calculations based on conserved hydrodynamic volume are expected to underestimate the viscosity radius of PEGylated proteins.

Figure 8 is indicating that the slope of increase in R_h_ of PEGylated proteins is much sharper than native standard proteins. Also from this figure can be understood that at the same molecular weight, PEGylated protein will have much bigger R_h_.

Studying the effect of polymer’s structure on purification profile of PEGylated proteins will be followed using various molecular weights of branched and linear PEG in future study.

**Figure 9:**
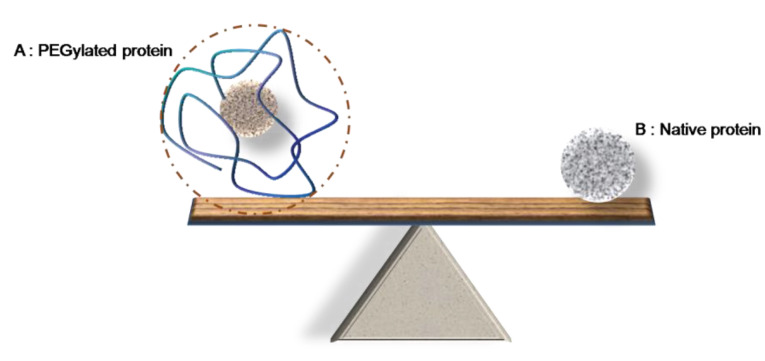
Schematic image representing the effect of PEGylation on protein size. (A) is representing the PEGylated human serum albumin by Mw of 66.5 kDa, which is PEGylated by 20 kDa linear PEG chain. (B) is the native protein by Mw equal to the sum of molecular weight of HSA + PEG (66.5 + 20 = 86.5 kDa).

Figure 9 is schematically summarizing the achieved data as after PEGylation while the Mw of protein increases by attaching the polymer, its hydrodynamic radius becomes much bigger than a native protein with the Mw equal to the sum of molecular weights of protein and attached PEG.

## Conclusion

At this study three different molecular weights of linear mPEG-maleimide and also 20 kDa branched mPEG-maleimide were applied. Increasing the size of attached PEG increases the resolution of purification in SEC method due to increased size of PEGylated protein, and in IExC method due to weaker interaction between column surface and protein as a result of steric hindrance imposes by attached PEG. As has discussed, increasing the PEG size, results in linear increase in the hydrodynamic radius of protein and doubles it by doubling the size of attached PEG.

Based on obtained results the higher value for the sum of R_h_ of PEG and R_h_ of protein, in compare to R_h_ value of PEGylated protein, could be explain the shielded attachment of PEG to protein versus linear attachment which has schematically shown at Figure 7b. PEG is hydrophobic and flexible that these two properties of it can explain this proposal.

In SDS-PAGE PEGylated HSA had relative mobility (R_f_) less than what was expected as its Mw is 85.5 kDa, while its R_f_ value was similar to a standard protein with Mw equal to approximately 95 kDa. This slower mobility of PEGylated form on gel happens because of the formation of a complex between PEG and SDS. Changes in the structure of attached PEG (linear or branched) did not show significant difference between hydrodynamic radius of modified proteins (Table 1), but slightly late elution of branched PEG-HSA in IExC compared to linear PEG-HSA (Figure 2b) might be as a result of different shielding effect of attached PEGs because of their different structures, that results in different surface charge and/or different steric hinderance they could impose.

It remains possible that a branched PEG-protein might have less conformational flexibility than a linear PEG-protein. Thus the effective sizes of two species while passing through a membrane pore may be different despite those are the same in free solution.

It should be mention that the data of this study was not enough to discuses more certainly about the effect of structure on elution profile of PEGylated protein.

According to achieved data it can be concluded that after PEGylation while the Mw of protein increases by attaching the polymer, its hydrodynamic radius becomes much bigger than a native protein with the Mw equal to the sum of molecular weights of protein and attached PEG.

## Conflict of Interest

The authors confirm that there is no conflict of interest.

## Acknowledgments

This work is supported by Drug Applied Research Center, and was supported by grant number 93/122. We also would like to express our appreciation to Professor Alois Jungbauer who kindly allowed us to conduct part of this research in his laboratory.
